# It is beneficial to invest resources to implement proton intracranial SRS

**DOI:** 10.1002/acm2.13701

**Published:** 2022-06-17

**Authors:** Rohan Deraniyagala, Xuanfeng Ding, Michelle Alonso‐Basanta, Taoran Li, Yi Rong

**Affiliations:** ^1^ Department of Radiation Oncology William Beaumont Hospital Royal Oak Michigan USA; ^2^ Department of Radiation Oncology University of Pennsylvania Philadelphia Pennsylvania USA; ^3^ Department of Radiation Oncology Mayo Clinic Arizona Phoenix Arizona USA

**Keywords:** brain radiation‐induced necrosis, proton beam therapy, stereotactic radiosurgery

## 1 INTRODUCTION

Radiation oncology is a unique field in medicine, where technological advancement is one of the main drivers for more precise treatment with less toxicities and better outcomes. Yet “technology constant evolving” might be a blessing and a curse at the same time for our field. It is a blessing as we have seen drastic survival benefits for cancer patients, in part thanks to the technological advancement,^1^ but it may also be a curse as the reported clinical outcomes may only be subject to the technology used at that time and may not be directly applied to treatments of new technology down the road. Proton‐based single or hypofractionated stereotactic radiosurgery (SRS) for treating skull base meningiomas (>0.2 and <4.0 cm) has been reported decades ago using proton technology at the time, passive scattering with two beam arrangement.^2^, ^3^, ^4^ Excellent local control outcomes with these studies indicated the strong efficacy of proton‐based SRS for single solid tumor. On the other hand, proton‐based single or fractionated SRS series for vestibular schwannoma reported excellent local control and cranial nerve preservation, compared to photon‐based SRS, yet associated with failure in hearing preservation rates.^5^, ^6^ A recent expert review concluded that proton beam therapy (PBT) had shown clinical indications for skull‐based tumors when there is a need for a high dose, with an appropriate tumor size, and the treatment location abutting several radiosensitive organs. Note that most of the studies cited in this review were based on proton and photon technology at the time, and the benefit of proton SRS remains unclear for brain metastases. Now that with further technological advancement in both proton and photon,^7^ *is it beneficial to invest resources to implement proton intracranial SRS?* Herein, we invited radiation oncologists and medical physicists experienced in photon and proton to touch on both clinical and technological aspects for this debate topic.

Arguments for the proposition are given by Dr. Rohan Deraniyagala and Dr. Xuanfeng Ding. Dr. Rohan Deraniyagala obtained his Bachelor of Science in computer engineering from the University of Michigan in Ann Arbor, MI. He attended Wayne State Medical School in Detroit, MI and went to the University of Florida in Gainesville, FL to complete his radiation oncology residency. Dr. Deraniyagala also completed a fellowship in proton therapy at the University of Florida Health Proton Therapy Institute in Jacksonville, FL. He is currently the clinical director of Beaumont Proton Therapy Institute and an assistant professor in radiation oncology at Oakland University William Beaumont School of Medicine. He has dozens of peer‐reviewed publications and presentations. His research interests are head‐and‐neck malignancies, pediatric oncology, and arc‐based proton therapy.

Dr. Xuanfeng Ding received his Ph.D. in Physics from Wake Forest University in 2012 and finished his residency training at the University of Pennsylvania in 2014. After commissioning the first PBS compact proton system in Willis‐Knighton Cancer Center, Dr. Ding joined William Beaumont Hospital, Royal Oak, MI in 2015, as the lead proton physicist and assistant professor. Dr. Ding's research interests include proton arc technique, adaptive therapy, and motion management. He received several extramural research grants as the PI and was granted multiple patents. Dr. Ding published over 40 peer‐reviewed papers and hundreds of conference abstracts. He is certified by the American Board of Radiology in Therapeutic Radiologic Physics. He served as the cochair in the European Society of Radiotherapy and Oncology physics workshop: Particle Arc Therapy in 2022, president of the Great Lakes Chapter AAPM in 2020, and committee member of several AAPM Task and Work Groups.

Arguments against the proposition are given by Dr. Michelle Alonso‐Basanta and Dr. Taoran Li. Dr. Michelle Alonso‐Basanta is a board‐certified Associate Professor of Radiation Oncology at the Perelman School of Medicine, University of Pennsylvania. She is the Vice‐Chair of Clinical Affairs and Section Chief for Central Nervous System Tumors in the Department of Radiation Oncology at Penn Medicine. She received her undergraduate degree in Chemistry from New York University (NYU) where she also completed her MD/Ph.D. She completed her residency in radiation oncology at NYU where she was Chief Resident. Dr. Alonso‐Basanta specializes in diseases of the central nervous system, the base of skull, head and neck, and spine, including expertise in stereotactic cranial and extracranial radiotherapy. She is trained in image‐guided, intensity‐modulated radiotherapy, proton radiotherapy, SRS (Gamma Knife radiosurgery), and stereotactic radiotherapy. Her clinical and research interests include normal tissue sparing and the prevention and management of long‐term effects in patients with central nervous system tumors.

Dr. Taoran Li is an Assistant Professor of Radiation Oncology and the Director of Medical Physics Residency at Penn. Prior to joining the University of Pennsylvania, he had worked at Thomas Jefferson University as an Associate Director of Medical Physics Residency and a member of the SRS services at Jefferson Hospital of Neuroscience. Dr. Li received his Ph.D. in Medical Physics from Duke University in 2013 and subsequently stayed at Duke to complete his residency in therapeutic medical physics before moving to Philadelphia. He has published over 40 peer‐reviewed journal articles and 2 book chapters on advanced and novel treatment delivery techniques and equipment, including linac‐based SRS, Halcyon, adaptive RT, and auto planning. His clinical and research expertise on SRS focuses on better characterizing different SRS planning and delivery platforms, including Gamma Knife, Elements, and HyperArc through multi‐institutional studies, and better standardizing SRS practices.

## 2 OPENING STATEMENT

### 2.1 For the proposition: Rohan Deraniyagala, M.D. and Xuanfeng Ding, Ph.D

#### 2.1.1 From a physician's point of view

Stratification for survival with brain metastases has improved over the last two decades. This is largely because of the ability to query large databases to develop robust prognostic scoring systems and improvements in treating metastatic disease.^8^ The efficacy of systemic therapy, including immunotherapy has improved overall survival in patients with brain metastases. Ipilimumab improved overall survival in a Phase III randomized trial of melanoma patients.^9^ Especially, it reported a prolonged survival with stereotactic body radiation therapy in the treatment of oligometasis.^10^ If such treatment of metastasis has improved overall survival, it is expected that the durable and safe treatment of brain metastasis would also offer prolonged survival.

From a clinical perspective, patients need efficient conformal treatment. Brain radiation–induced necrosis (RN) is the most common toxicity from SRS. This can result in temporary or permanent neurological damage. Treatment for this involves corticosteroids, Avastin, or hyperbaric oxygen. These additional therapies can delay or interfere with cancer‐directed treatments aimed at preserving a patient's survival.

Previous studies have suggested that the V12Gy of normal brain tissue is predictive of RN.^11^, ^12^ Additionally, the dose to the hippocampus is also associated with a cognitive function decrease.^13^, ^14^ Thus, continuing to invest and develop superior treatment technologies that can offer a better dosimetric outcome shall be the direction and commitment of health‐care professionals and researchers. PBT has shown significant potential for SRS because of its intrinsic dosimetry advantages over photon radiotherapy with the rotational arc concept.^15^ Though it may take years to develop and implement such a concept in clinical practice, I am optimistic that this technological revolution will reshape the role of PBT.

#### 2.1.2 From a physicist's point of view

It is always worth investing in and developing superior techniques for unmet clinical needs. Aside from emerging clinical evidence, one of the driving factors shifting from whole brain radiotherapy (WBRT) toward intracranial SRS is the technological advancements in photon radiotherapy.^16^ In the last decades, we experienced a series of technological revolutions in imaging, planning, and treatment such as on‐broad cone‐beam CT (CBCT),^17^ volumetric modulated arc therapy (VMAT)^18^. These new improvements and broad clinical implementations built a solid foundation for the wide adoption of intracranial SRS‐based photon radiotherapy even for patients with multiple brain metastases.

On the other hand, technology development in proton therapy has been lacking in this aspect. There are few reports on the use of proton SRS for brain metastases.^19^ Admittedly, with the current PBS system, the PBT may not be comparable to photon radiotherapy in terms of lateral penumbra,^20^ target conformity,^21^ and uncertainties,^22^ which are critical for SRS. However, PBT has its dosimetry advantages utilizing the “Bragg peak,” which has not been fully explored in the area of intracranial SRS.

In my opinion, there are several areas worth investigating toward proton SRS:

1. New treatment technologies that enable shaper dose fall‐off for the PBS nozzle:

haper dose fall‐off is the first prerequisite for clinical proton SRS implementation, mitigating the risk of RN. Recently, two research directions showed potential (a) dynamic collimation system,^23^ which effectively sharpens the lateral penumbra in the peripheral intracranial targets; and (b) proton arc therapy,^24^ which showed better dosimetric quality in terms of target conformity and V12Gy to the brain tissue compared to conventional VMAT plans, especially for large targets that may not be qualified for single fractionation photon SRS due to potential toxicity.^8^

1. Comprehensive IGRT systems that enable pretreatment dose validation:

Uncertainties are inherent with PBT, mostly from CT (3.5% range uncertainty), patient's geometry changes, and setup errors. Without an easily accessible and reliable pretreatment validation tool, such uncertainties pose a risk for proton SRS. Several imaging technologies may mitigate the issue: (a) CT‐on‐rail,^25^ and (b) synthetic CT created from daily CBCT for proton dose calculation.^26^, ^27^ These 3D volumetric images could offer clinicians, therapists, and physicists valuable dose reconstruction information for the optimal decision on the treatment day.

1. Re‐irradiation of brain SRS:

RN is one of the major concerns in re‐irradiation SRS. Unpredictable adverse radiation effects also make repeat SRS an unpalatable option.^28^ PBT may open the door for repeat treatments, considering its advantage of a less integral dose.

In summary, there are clinical indications of a better intracranial SRS program for larger targets and re‐irradiation treatments, thus, investing resources in new treatment and imaging techniques for proton SRS programs is necessary for those clinical indications.

### 2.2 Against the proposition: Michelle Alonso‐Basanta M.D., Ph.D. and Taoran Li, Ph.D

As more cancer patients live longer, intracranial SRS has been increasingly utilized to manage recurrent brain metastases, offering patients lower cognitive toxicity and additional opportunity for novel therapies such as immunotherapy and novel chemotherapy agents.^29^ Most recent clinical trials and studies have shown that multi‐target SRS is a safe and highly effective treatment for brain metastasis with much reduced cognitive toxicity compared to WBRT.^30^ Although proton SRS may offer some hypothetical dosimetric benefit,^31^ we argue that currently it is not beneficial to invest substantial resources to implement proton SRS—rather, these resources should be devoted to improve and standardize current photon‐based SRS practice.

#### 2.2.1 SRS accessibility and cost‐effectiveness

We can all agree that radiosurgery is a highly effective, noninvasive treatment that improves the patient quality of life. Yet the utilization of radiosurgery is still limited and largely nonuniform.^32^ Although proton therapy is being offered at an increasing number of clinics, it is still a scarce and expensive resource, compared to existing linac‐ and Gamma Knife–based practices. As we see more patients who would benefit from SRS treatment for the longitudinal management of brain metastasis, more resources should be devoted to increase the accessibility of linac‐ and Gamma Knife–based SRS through personnel training and practice standardization, and to implement novel, more affordable SRS solutions (such as ZAP‐X), as opposed to develop proton SRS programs that are substantially limited to only a few centers, much more expensive to operate, and with lower insurance approval rate.^33^

#### 2.2.2 Clinical benefit of proton SRS is unclear

At this time, there are no clinical trials comparing photon‐based stereotactic modalities with proton SRS. Only one large series retrospective review noted the feasibility and overall outcome of these patients.^19^ In reality, many patients receiving SRS for brain metastasis are being treated to multiple targets at the same time. Although limited literature has shown that proton SRS is technically feasible,^34^ their comparison to the photon counterpart did not reflect the modern SRS technolog using photons.

#### 2.2.3 Physical limitations of proton treatment make it suboptimal for SRS

Proton treatment has inherent limitations and uncertainties associated with the physical process of proton production, transport, modulation, and interaction with the media, which makes it a suboptimal candidate for ultra‐high‐precision targeted treatment. Range, spot size, and linear energy transport (LET) uncertainties, all pose spatially sensitive dosimetric variations on the order of several millimeters that are difficult to model. At the same time, submillimeter spatial accuracy is one, if not the most, critical aspect of successful SRS treatment when we target tumors less than 5 mm in diameter. Boczkowski et al. compared proton SRS with state‐of‐the‐art photon SRS using the HyperArc platform for a single target and concluded that proton is inferior in terms of plan quality and delivery uncertainties.^34^ The best use of proton is for large targets adjacent to critical parallel organs, as opposed to small targets next to serial organs, which is very typical for cranial radiosurgery.

#### 2.2.4 Better standardization of photon SRS is urgently needed

As we see the increased utilization of photon‐based SRS in free‐standing and community clinics, substantial variation still exists among treatment teams. These variations include hardware and software variations, planners with different experience levels, lack of consensus margin, and motion uncertainty management guidelines. Moreover, single‐isocenter multi‐target SRS treatment has seen substantial utilization yet without well‐understood uncertainty management, toxicity/outcome data, and practice guidelines. Vergalasova et al. demonstrated that substantial trade‐offs among plan quality, delivery efficiency, and staff resources variations existed among different treatment techniques, and across human SRS planners using the same technique.^35^ All of the above mentioned areas urgently await improvements, which require a significant amount of community resources and support. Therefore, our limited resources should be devoted to first better standardize photon‐based SRSs that are directly impacting more patient treatments today.

In summary, SRS treatment is safe and effective, but its accessibility and standardization still need substantial and urgent improvement. Although proton therapy has its unique advantage and indications for many disease sites, its application in SRS has limited clinical evidence, and the delivery accuracy is inherently limited due to the first‐principle‐driven spatial uncertainty of proton beams that is difficult to overcome. Therefore, at this stage, we should not devote significant resource in implementing proton SRS; instead, the community should focus on how to better utilize existing treatment modalities to provide high‐quality SRS to more patients with better standardization.

## 3 REBUTTAL

### 3.1 For the proposition: Rohan Deraniyagala, M.D. and Xuanfeng Ding, Ph.D

Dr. Li and Dr. Alonso‐Basanta made an excellent point regarding the accessibility and justification of utilizing expensive PBT for intracranial radiosurgery. This remains an issue as we have little clinical evidence.^36^ On the other hand, these challenges in balancing the treatment outcome and cost reside in every aspect of our lives and our clinical practice, not limited to intracranial radiosurgery.^37^, ^38^ Fortunately, things started changing recently. A recent study found that PBT lowers the risk of grade 4 lymphopenia compared with photon in esophageal cancer patients in which severe lymphopenia is correlated to inferior treatment outcomes.^39^ A similar finding was reported in a Phase II randomized glioblastoma study.^40^ Moreover, a multi‐institutional Phase II study showed PBT has a high rate of local control for localized, unresectable hepatocellular carcinoma and intrahepatic cholangiocarcinoma.^41^ This emerging clinical evidence is encouraging, and it demonstrates that the dosimetric advantage can lead to a clinical benefit with the intracranial SRS using PBT.

In terms of the dosimetric plan quality for SRS, it has been a challenging topic for the entire proton therapy society,^20^ but we need to point out that proton therapy planning and delivery technology continues to evolve.^23^, ^24^ The dosimetric comparison study mentioned in the opening statement between proton versus HyperArc was based on a passive‐scattering system that may not represent the outcome of the most advanced proton treatment techniques.^34^ SRS’ precision seems to be incompatible with the uncertainties associated with PBT, such as range, LET, and motion. However, the recent development of the spot‐scanning proton arc (SPArc) therapy^21^ may help mitigate such concerns. Previous studies demonstrated the improved dosimetric robustness for spine SRS in the presence of geometry change,^42^ interplay effect mitigation in the lung SRBT,^43^ and the capability of LET optimization.^44^ Furthermore, the recent paper showed a dosimetric advantage in single brain metastases utilizing SPArc compared to VMAT, which is encouraging.^15^

On the other hand, balancing health‐care resources and maximizing clinical benefits is important for our society. Standardization and accessibility to intracranial SRS are critical, but they should apply to both photon and proton in terms of planning, treatment delivery, and patient selection. We are glad to see the tremendous progress and a broader adoption across the European countries utilizing the Dutch Model for proton patient selection.^45^ A similar approach can be applied to intracranial SRS to optimize the health‐care resource for patients in the United States. For example, based on the NTCP model, the brain metastases patients who may benefit most from SPArc are the large targets that are not qualified or safe for the single‐fraction photon SRS.^15^ This patient population will directly benefit from PBT because of the lowered risk of RN defined by the V12Gy volume.

At last, any innovation and improvement cost tremendous financial investments, courage, and time commitment. However, these burdens and challenges should not limit our communities’ imagination from making innovative ideas and technological improvements to benefit our patient's quality of life who need better intracranial SRS treatment. So, when we access potential clinical benefits and opportunities against the extra cost and resources for intracranial SRS, let us be patient, persistent, and creative.

### 3.2 Against the proposition: Michelle Alonso‐Basanta M.D., Ph.D. and Taoran Li, Ph.D

We thank Drs. Deraniyagala and Ding for their excellent opening statement. We agree with the need to better understand proton therapy's role in intracranial SRS, particularly its applications in re‐irradiation settings. We also share that exciting potential technical advancements could help overcome some first‐principle‐driven challenges that proton beams face compared to photon therapy.

From the clinical perspective, there is no question that the evolution of systemic therapy over the last few years has provided prolonged survival in a population that had previously been deemed “palliative.” In addition, I agree that the conformality of treatment is important as less brain receiving any radiation dose decreases the risk of RN. Although I appreciate the role that SPArc can provide, this has only been modeled for single lesions. However, we are no longer discussing the value of treating patients with less than 3 metastases for patients. Many times, we are discussing more than 8–10 lesions in one or multiple sessions, and therefore it is important that we review the dosimetric means by which we can treat multiple targets with protons when we have a multitude of photon modalities that can be used more than once to treat patients over the course of their disease management. In addition, as we explore the role of proton FLASH for the treatment of a number of malignancies,^46^, ^47^ does it make sense to spend money and time on a technique that may become obsolete before it can take off?

From the technical perspective, it is indeed very exciting to see that novel proton delivery technologies such as dynamic collimator and proton arc therapy are entering the clinical realm to help overcome several technical challenges with the current proton therapy delivery for SRS applications. However, it is uncertain how much these technologies will help when the physical interaction of proton particles with beam degrader, beam transport, and patient anatomy carries inherent stochastic interaction. This stochastic interaction is likely to limit the best achievable accuracy of proton beams to 1–2 mm, which is substantially larger than photon delivery system accuracy (∼0.5 mm) and can be highly relevant when targeting brain metastasis with diameters <3 mm.

In addition to uncertainties associated with the physics aspect of proton beam transport and interaction, substantial uncertainty also exists in the radiobiology aspect of proton beam, particularly due to LET uncertainty at the end of the Bragg peak. The current proton planning system uses a simplified relative‐biological effectiveness (RBE) value of 1.1. It has been shown that actual RBE varies depending on tissue type, dose levels, and LET.^48^ More importantly, due to the LET variation along the proton pencil beam track, the RBE at the distal end of the Bragg peak is likely higher than 1.1 but with large uncertainty due to lack of consensus in RBE estimation. This is particularly true for low energy beams with small spread out Bragg peak width,^49^ which is exactly the type of beam needed for cranial SRS. Currently, no commercially available treatment planning system is capable of accurately estimate RBE for small field, and the stake of having an uncertain yet elevated local dose distal to a very small target can be very high for proton SRS treatment.

As earlier mentioned, we agree that proton therapy for relatively large cranial targets remains a very valuable treatment option, particularly in the context of re‐irradiation. However, because of the physical and radio‐biological limitations associated with proton treatment, photon modalities retain competitive advantage over proton beam in treating small cranial targets. Although we should certainly continue to explore and push the envelope of proton delivery techniques and radiobiology research, implementing a clinical proton SRS program may not be the most optimal way of spending their resources for the vast majority of clinics and patients requiring SRS. Just like an old saying, one should not run before one can walk. At the same time, novel proton treatment such as FLASH techniques may soon demonstrate its advantage over proton SRS, particularly when targeting multiple lesions at the same time.

## CONFLICT OF INTEREST

The author declares that there is no conflict of interest that could be perceived as prejudicing the impartiality of the research reported. T.L. reported honororia, travel expenses, and research grant from Varian Medical Systems unrelated to the work presented. X.D. reported honorarium from IBA and Elekta speaker Bureau and research fundings from IBA, Elekta and RadioMed outside the work presented.

## References

[1] Bryant AK , Banegas MP , Martinez ME , et al. Trends in radiation therapy among cancer survivors in the United States, 2000–2030. Cancer Epidemiol Biomarkers Prev. 2017;26:963‐970. 28096199 10.1158/1055-9965.EPI-16-1023

[2] Gudjonsson O , Blomquist E , Nyberg G , et al. Stereotactic irradiation of skull base meningiomas with high energy protons. Acta Neurochir (Wien). 1999;141:933‐940. 10526074 10.1007/s007010050399

[3] Vernimmen FJ , Harris JK , Wilson JA , et al. Stereotactic proton beam therapy of skull base meningiomas. Int J Radiat Oncol Biol Phys. 2001;49:99‐105. 11163502 10.1016/s0360-3016(00)01457-7

[4] Halasz LM , Bussiere MR , Dennis ER , et al. Proton stereotactic radiosurgery for the treatment of benign meningiomas. Int J Radiat Oncol Biol Phys. 2011;81:1428‐1435. 20934263 10.1016/j.ijrobp.2010.07.1991

[5] Harsh GR , Thornton AF , Chapman PH , et al. Proton beam stereotactic radiosurgery of vestibular schwannomas. Int J Radiat Oncol Biol Phys. 2002;54:35‐44. 12182972 10.1016/s0360-3016(02)02910-3

[6] Weber DC , Chan AW , Bussiere MR , et al. Proton beam radiosurgery for vestibular schwannoma: tumor control and cranial nerve toxicity. Neurosurgery. 2003;53:577‐586. discussion 586‐578. 12943574 10.1227/01.neu.0000079369.59219.c0

[7] Hyer DE , Ding XF , Rong Y . Proton therapy needs further technological development to fulfill the promise of becoming a superior treatment modality (compared to photon therapy). J Appl Clin Med Phys. 2021;22:4‐11. 10.1002/acm2.13450 PMC8598137 34730268

[8] Chan JL , Lee SW , Fraass BA , et al. Survival and failure patterns of high‐grade gliomas after three‐dimensional conformal radiotherapy. J Clin Oncol. 2002;20:1635‐1642. 11896114 10.1200/JCO.2002.20.6.1635

[9] Margolin K , Ernstoff MS , Hamid O , et al. Ipilimumab in patients with melanoma and brain metastases: an open‐label, phase 2 trial. Lancet Oncol. 2012;13:459‐465. 22456429 10.1016/S1470-2045(12)70090-6

[10] Otake S , Goto T . Stereotactic radiotherapy for oligometastasis. Cancers (Basel). 2019;11:133. 10.3390/cancers11020133 PMC6407034 30678111

[11] Minniti G , Clarke E , Lanzetta G , et al. Stereotactic radiosurgery for brain metastases: analysis of outcome and risk of brain radionecrosis. Radiat Oncol. 2011;6:48. 21575163 10.1186/1748-717X-6-48 PMC3108308

[12] Blonigen BJ , Steinmetz RD , Levin L , et al. Irradiated volume as a predictor of brain radionecrosis after linear accelerator stereotactic radiosurgery. Int J Radiat Oncol Biol Phys. 2010;77:996‐1001. 19783374 10.1016/j.ijrobp.2009.06.006

[13] Marsh JC , Gielda BT , Herskovic AM , Abrams RA . Cognitive sparing during the administration of whole brain radiotherapy and prophylactic cranial irradiation: current concepts and approaches. J Oncol. 2010;2010:198208. 20671962 10.1155/2010/198208 PMC2910483

[14] Raber J , Rola R , LeFevour A , et al. Radiation‐induced cognitive impairments are associated with changes in indicators of hippocampal neurogenesis. Radiat Res. 2004;162:39‐47. 15222778 10.1667/rr3206

[15] Chang S , Liu G , Zhao L , et al. Redefine the role of spot‐scanning proton beam therapy for the single brain metastasis stereotactic radiosurgery. Front Oncol. May 2022. 10.3389/fonc.2022.804036 PMC9160604 35664795

[16] Li J , Brown PD . The diminishing role of whole‐brain radiation therapy in the treatment of brain metastases. JAMA Oncol. 2017;3:1023‐1024. 28056128 10.1001/jamaoncol.2016.5411

[17] Jaffray DA , Siewerdsen JH , Wong JW , Martinez AA . Flat‐panel cone‐beam computed tomography for image‐guided radiation therapy. Int J Radiat Oncol Biol Phys. 2002;53:1337‐1349. 12128137 10.1016/s0360-3016(02)02884-5

[18] Yu CX , Li XA , Ma LJ , et al. Clinical implementation of intensity‐modulated arc therapy. Int J Radiat Oncol Biol Phys. 2002;53:453‐463. 12023150 10.1016/s0360-3016(02)02777-3

[19] Atkins KM , Pashtan IM , Bussiere MR , et al. Proton stereotactic radiosurgery for brain metastases: a single‐institution analysis of 370 patients. Int J Radiat Oncol Biol Phys. 2018;101:820‐829. 29976494 10.1016/j.ijrobp.2018.03.056

[20] Wang DX , Dirksen B , Hyer DE , et al. Impact of spot size on plan quality of spot scanning proton radiosurgery for peripheral brain lesions. Med Phys. 2014;41:121705. 25471952 10.1118/1.4901260

[21] Ding X , Li X , Qin A , et al. Have we reached proton beam therapy dosimetric limitations? – A novel robust, delivery‐efficient and continuous spot‐scanning proton arc (SPArc) therapy is to improve the dosimetric outcome in treating prostate cancer. Acta Oncol. 2018;57:435‐437. 28774218 10.1080/0284186X.2017.1358463

[22] Lomax AJ . Intensity modulated proton therapy and its sensitivity to treatment uncertainties 2: the potential effects of inter‐fraction and inter‐field motions. Phys Med Biol. 2008;53:1043‐1056. 18263957 10.1088/0031-9155/53/4/015

[23] Hyer DE , Hill PM , Wang D , et al. A dynamic collimation system for penumbra reduction in spot‐scanning proton therapy: proof of concept. Med Phys. 2014;41:091701. 25186376 10.1118/1.4837155

[24] Ding X , Li X , Zhang JM , et al. Spot‐scanning proton arc (SPArc) therapy: the first robust and delivery‐efficient spot‐scanning proton arc therapy. Int J Radiat Oncol Biol Phys. 2016;96:1107‐1116. 27869083 10.1016/j.ijrobp.2016.08.049

[25] Nesteruk KP , Bobic M , Lalonde A , et al. CT‐on‐rails versus in‐room CBCT for online daily adaptive proton therapy of head‐and‐neck cancers. Cancers (Basel). 2021;13:5991. 34885100 10.3390/cancers13235991 PMC8656713

[26] Qin A , Chen S , Liu G , et al. The feasibility and accuracy of utilizing CBCT and generative‐adversarial‐network (GAN) to perform proton treatment dose evaluation for lung and head and neck patients. Int J Radiat Oncol Biol Phys. 2020;108:S41‐S42.

[27] Thummerer A , Zaffino P , Meijers A , et al. Comparison of CBCT based synthetic CT methods suitable for proton dose calculations in adaptive proton therapy. Phys Med Biol. 2020;65:095002. 32143207 10.1088/1361-6560/ab7d54

[28] Mckay WH , McTyre ER , Okoukoni C , et al. Repeat stereotactic radiosurgery as salvage therapy for locally recurrent brain metastases previously treated with radiosurgery. J Neurosurg. 2017;127:148‐156. 27494815 10.3171/2016.5.JNS153051

[29] Chang EL , Wefel JS , Hess KR , et al. Neurocognition in patients with brain metastases treated with radiosurgery or radiosurgery plus whole‐brain irradiation: a randomised controlled trial. Lancet Oncol. 2009;10:1037‐1044. 19801201 10.1016/S1470-2045(09)70263-3

[30] Serizawa T , Yamamoto M , Higuchi Y , et al. Local tumor progression treated with Gamma Knife radiosurgery: differences between patients with 2–4 versus 5–10 brain metastases based on an update of a multi‐institutional prospective observational study (JLGK0901). J Neurosurg. 2019;132:1480‐1489. 31026833 10.3171/2019.1.JNS183085

[31] Lin MH , Yang M , Dougherty J , et al. Radiation therapy for pediatric brain tumors using robotic radiation delivery system and intensity modulated proton therapy. Pract Radiat Oncol. 2020;10:e173‐e182. 31542454 10.1016/j.prro.2019.09.008

[32] Pannullo SC , Julie DAR , Chidambaram S , et al. Worldwide access to stereotactic radiosurgery. World Neurosurg. 2019;130:608‐614. 31581410 10.1016/j.wneu.2019.04.031

[33] Kirkpatrick JP , Laack NN , Halasz LM , et al. Proton therapy for brain metastases: a question of value. Int J Radiat Oncol Biol Phys. 2018;101:830‐832. 29976495 10.1016/j.ijrobp.2018.05.005

[34] Boczkowski A , Kelly P , Meeks SL , et al. Proton vs Hyperarc radiosurgery: a planning comparison. J Appl Clin Med Phys. 2020;21:96‐108. 10.1002/acm2.13075 PMC7769415 33151014

[35] Vergalasova I , Liu H , Alonso‐Basanta M , et al. Multi‐institutional dosimetric evaluation of modern day stereotactic radiosurgery (SRS) treatment options for multiple brain metastases. Front Oncol. 2019;9:483. 31231614 10.3389/fonc.2019.00483 PMC6568036

[36] Allen AM , Pawlicki T , Dong L , et al. An evidence based review of proton beam therapy: the report of ASTRO's emerging technology committee. Radiother Oncol. 2012;103:8‐11. 22405807 10.1016/j.radonc.2012.02.001

[37] Brodin NP , Kabarriti R , Schechter CB , et al. Individualized quality of life benefit and cost‐effectiveness estimates of proton therapy for patients with oropharyngeal cancer. Radiat Oncol. 2021;16:19. 33478544 10.1186/s13014-021-01745-1 PMC7819210

[38] Smith WL , Smith CD , Patel S , et al. What conditions make proton beam therapy financially viable in Western Canada?. Cureus. 2018;10:e3644. 30723643 10.7759/cureus.3644 PMC6351082

[39] Shiraishi Y , Fang P , Xu C , et al. Severe lymphopenia during neoadjuvant chemoradiation for esophageal cancer: a propensity matched analysis of the relative risk of proton versus photon‐based radiation therapy. Radiother Oncol. 2018;128:154‐160. 29248170 10.1016/j.radonc.2017.11.028 PMC5999560

[40] Mohan R , Liu AY , Brown PD , et al. Proton therapy reduces the likelihood of high‐grade radiation‐induced lymphopenia in glioblastoma patients: phase II randomized study of protons vs photons. Neuro‐oncol. 2021;23:284‐294. 32750703 10.1093/neuonc/noaa182 PMC7906048

[41] Hong TS , Wo JY , Yeap BY , et al. Multi‐institutional Phase II study of high‐dose hypofractionated proton beam therapy in patients with localized, unresectable hepatocellular carcinoma and intrahepatic cholangiocarcinoma. J Clin Oncol. 2016;34:460‐468. 26668346 10.1200/JCO.2015.64.2710 PMC4872014

[42] Liu G , Li XQ , Qin A , et al. Is proton beam therapy ready for single fraction spine SBRS? – A feasibility study to use spot‐scanning proton arc (SPArc) therapy to improve the robustness and dosimetric plan quality. Acta Oncol (Madr). 2021;60:653‐657. 10.1080/0284186X.2021.1892183 33645429

[43] Liu G , Zhao L , Qin A , et al. Lung stereotactic body radiotherapy (SBRT) using spot‐scanning proton arc (SPArc) therapy: a feasibility study. Front Oncol. 2021;11:664455. 33968770 10.3389/fonc.2021.664455 PMC8100671

[44] Li X , Ding X , Zheng W , et al. Linear energy transfer incorporated spot‐scanning proton arc therapy optimization: a feasibility study. Front Oncol. 2021;11:698537. 34327139 10.3389/fonc.2021.698537 PMC8313436

[45] Langendijk JA , Hoebers FJP , de Jong MA , et al. National protocol for model‐based selection for proton therapy in head and neck cancer. Int J Part Ther. 2021;8:354‐365. 34285961 10.14338/IJPT-20-00089.1 PMC8270079

[46] Diffenderfer ES , Verginadis II , Kim MM , et al. Design, implementation, and in vivo validation of a novel proton FLASH radiation therapy system. Int J Radiat Oncol Biol Phys. 2020;106:440‐448. 31928642 10.1016/j.ijrobp.2019.10.049 PMC7325740

[47] Vorhees CV , Vatner RE , Williams MT . Review of conventional and high dose rate brain radiation (FLASH): neurobehavioural, neurocognitive and assessment issues in rodent models. Clin Oncol (R Coll Radiol). 2021;33:e482‐e491. 34548203 10.1016/j.clon.2021.09.002 PMC10114147

[48] Paganetti H , van Luijk P . Biological considerations when comparing proton therapy with photon therapy. Semin Radiat Oncol. 2013;23:77‐87. 23473684 10.1016/j.semradonc.2012.11.002

[49] Paganetti H , Blakely E , Carabe‐Fernandez A , et al. Report of the AAPM TG‐256 on the relative biological effectiveness of proton beams in radiation therapy. Med Phys. 2019;46:e53‐e78. 30661238 10.1002/mp.13390 PMC9559855

